# Supplementary material for: Effect of Subthalamic Nucleus Deep Brain Stimulation (STN-DBS) on balance performance in Parkinson's disease
Source: PLoS One. 2020 Sep 11;15(9):e0238936. doi: 10.1371/journal.pone.0238936 (PMC7486080; doi:10.1371/journal.pone.0238936)
Supplement: S2 Checklist — (DOCX) [file pone.0238936.s002.docx]

STROBE Statement—checklist of items that should be included in reports of observational studies

|  | Item No. | Recommendation | Page  No. | Relevant text from manuscript |
| --- | --- | --- | --- | --- |
| **Title and abstract** | 1 | (*a*) Indicate the study’s design with a commonly used term in the title or the abstract | Page 1 |  |
|  |  | (*b*) Provide in the abstract an informative and balanced summary of what was done and what was found | Page 1,line 11- Page 2,line 32 | STN-DBS could improve the performance(MDS-UPDRS 3.12, Berg Balance Scale BBS) and the Limits of Stability of PD patients in H&Y3 |
| Introduction | | | |  |
| Background/rationale | 2 | Explain the scientific background and rationale for the investigation being reported | Page 2,line 35- Page 2,line 42 | Primary Parkinson's disease (PD) is a common…. and even life safety |
| Objectives | 3 | State specific objectives, including any prespecified hypotheses | Page 2,line 43- Page 2,line 46 | Until now…verify the effect of different treatment. |
| Methods | | | |  |
| Study design | 4 | Present key elements of study design early in the paper | Page 2,line 49- Page 2,line53 | …were retrospective analyzed in the study |
| Setting | 5 | Describe the setting, locations, and relevant dates, including periods of recruitment, exposure, follow-up, and data collection | Page 2,line 48- Page 2,line 62 | Subjects…Inclusion criteria… |
| Participants | 6 | (*a*) *Cohort study*—Give the eligibility criteria, and the sources and methods of selection of participants. Describe methods of follow-up  *Case-control study*—Give the eligibility criteria, and the sources and methods of case ascertainment and control selection. Give the rationale for the choice of cases and controls  *Cross-sectional study*—Give the eligibility criteria, and the sources and methods of selection of participants | Page 2,line 48- Page 2,line 62 | Subjects…Inclusion criteria…Inclusion criteria and Exclusion criteria |
|  |  | (*b*) *Cohort study*—For matched studies, give matching criteria and number of exposed and unexposed  *Case-control study*—For matched studies, give matching criteria and the number of controls per case |  |  |
| Variables | 7 | Clearly define all outcomes, exposures, predictors, potential confounders, and effect modifiers. Give diagnostic criteria, if applicable |  |  |
| Data sources/ measurement | 8* | For each variable of interest, give sources of data and details of methods of assessment (measurement). Describe comparability of assessment methods if there is more than one group | Page 4,line 105- Page 5,line 126 | Evaluation of quality of life, motor disability, balance performance, and limit of stability…Statistical analyses… |
| Bias | 9 | Describe any efforts to address potential sources of bias |  |  |
| Study size | 10 | Explain how the study size was arrived at |  |  |

Continued on next page

| Quantitative variables | 11 | Explain how quantitative variables were handled in the analyses. If applicable, describe which groupings were chosen and why |  |  |
| --- | --- | --- | --- | --- |
| Statistical methods | 12 | (*a*) Describe all statistical methods, including those used to control for confounding | Page 5,line 127- Page 5,line 130 | Statistical analysis… |
|  |  | (*b*) Describe any methods used to examine subgroups and interactions |  |  |
|  |  | (*c*) Explain how missing data were addressed |  |  |
|  |  | (*d*) *Cohort study*—If applicable, explain how loss to follow-up was addressed  *Case-control study*—If applicable, explain how matching of cases and controls was addressed  *Cross-sectional study*—If applicable, describe analytical methods taking account of sampling strategy |  |  |
|  |  | (*e*) Describe any sensitivity analyses |  |  |
| Results | | | | |
| Participants | 13* | (a) Report numbers of individuals at each stage of study—eg numbers potentially eligible, examined for eligibility, confirmed eligible, included in the study, completing follow-up, and analysed | Page 5,line 132- Page 6,line 138 | We found no differences… |
|  |  | (b) Give reasons for non-participation at each stage |  |  |
|  |  | (c) Consider use of a flow diagram |  |  |
| Descriptive data | 14* | (a) Give characteristics of study participants (eg demographic, clinical, social) and information on exposures and potential confounders | Page 4,line 105- Page 5,line 126 | Motor disability (MDS-UPDRS III) and balance performance (MDS-UPDRS 3.12, BBS, LOS) were assessed at different times… |
|  |  | (b) Indicate number of participants with missing data for each variable of interest |  |  |
|  |  | (c) *Cohort study*—Summarise follow-up time (eg, average and total amount) |  |  |
| Outcome data | 15* | *Cohort study*—Report numbers of outcome events or summary measures over time | Page 6,line 139- Page 6,line 146; Page 12,line 297- Page 14,line 308 | Motor disability, balance performance(Tables 2, and 3) , and LoS… |
|  |  | *Case-control study—*Report numbers in each exposure category, or summary measures of exposure |  |  |
|  |  | *Cross-sectional study—*Report numbers of outcome events or summary measures |  |  |
| Main results | 16 | (*a*) Give unadjusted estimates and, if applicable, confounder-adjusted estimates and their precision (eg, 95% confidence interval). Make clear which confounders were adjusted for and why they were included |  |  |
|  |  | (*b*) Report category boundaries when continuous variables were categorized |  |  |
|  |  | (*c*) If relevant, consider translating estimates of relative risk into absolute risk for a meaningful time period |  |  |

Continued on next page

| Other analyses | 17 | Report other analyses done—eg analyses of subgroups and interactions, and sensitivity analyses |  |  |
| --- | --- | --- | --- | --- |
| Discussion | | | | |
| Key results | 18 | Summarise key results with reference to study objectives | Page 7,line 168- Page 7,line 170; Page 8,line 205- Page 8,line 214 | In this study, PDQ-39, motor disability, LoS, and LEDD immediately improved postoperation…  Dynamic adjustment of the static standing posture balance… |
| Limitations | 19 | Discuss limitations of the study, taking into account sources of potential bias or imprecision. Discuss both direction and magnitude of any potential bias | Page 9,line 222- Page 9,line 224 | …though the long-term effect on balance function in PD still requires further study |
| Interpretation | 20 | Give a cautious overall interpretation of results considering objectives, limitations, multiplicity of analyses, results from similar studies, and other relevant evidence | Page 6,line 148- Page 9,line 224 | In this study, the LEDD, PDQ-39 score, motor disability, and balance function…bias of the balance function score |
| Generalisability | 21 | Discuss the generalisability (external validity) of the study results | Page 9,line 222 | STN -DBS is widely used in treatment of PD… |
| Other information | |  | | |
| Funding | 22 | Give the source of funding and the role of the funders for the present study and, if applicable, for the original study on which the present article is based |  |  |

*Give information separately for cases and controls in case-control studies and, if applicable, for exposed and unexposed groups in cohort and cross-sectional studies.

**Note:** An Explanation and Elaboration article discusses each checklist item and gives methodological background and published examples of transparent reporting. The STROBE checklist is best used in conjunction with this article (freely available on the Web sites of PLoS Medicine at http://www.plosmedicine.org/, Annals of Internal Medicine at http://www.annals.org/, and Epidemiology at http://www.epidem.com/). Information on the STROBE Initiative is available at www.strobe-statement.org.
