# Supplementary material for: Effect of Subthalamic Nucleus Deep Brain Stimulation (STN-DBS) on balance performance in Parkinson's disease
Source: PLoS One. 2020 Sep 11;15(9):e0238936. doi: 10.1371/journal.pone.0238936 (PMC7486080; doi:10.1371/journal.pone.0238936)
Supplement: S1 File — (DOCX) [file pone.0238936.s003.docx]

Supplement:

PDQ-39：a questionnaire designed specifically for PD patients，it contains 8 parts: mobility, activities of daily living, emotional well-being, stigma, social support, cognition, communication, bodily discomfort. The higher score means worse quality of life.

MDS-UPDRS III：For motor function testing, it includes 18 questions. med-off的评分。The evaluation refers to the functional state of the patient who is currently taking anti-Parkinson drugs with the best efficacy status. Among them, the score of the balance related item is MDS-UPDRS 3.12: used to evaluate the patient's postural stability. During the assessment, the patient should stand up straight with eyes open, feet should be properly separated, and stand parallel. After the evaluator followed the patient's shoulder quickly and forcefully, he evaluated the stability of his posture by observing the patient's back reaction to the sudden shift of the body. The evaluation criteria are: 0 points, normal: no problem, stand back one or two steps back; 1 point, slight: need to back three to five steps, but do not need help from others to resume standing; 2 points, mild: need to back five Steps or more, but still does not need help from others to resume standing; 3 points, moderate: stand safely, but lacks postural balance reflex, if the scorer does not catch it will fall; 4 points, severe: posture is very unstable, inclined fall when lose balance spontaneously or touch shoulders slightly.

BBS：The Berg Balance Scale (BBS) measures balance in older people with impairment in balance function by assessing their performance on a number of functional motor tasks. It is a valid and reliable scale used in clinical practice and research. A higher score is associated with a lower fall risk. The total score ranges from 0 to 56. 0-20 points means poor balance function, 21-40 points means a certain balance ability, but people can walk with assistance, 41-56 means balance function is ok, people can walk independently, less than 40 points suggest there is a risk of falling.

TecnoBody PROKIN system was used to evaluate limit of stability of PD patients. The limit of stability (LoS) is the maximum displacement of the body mass center (COM) in all directions while standing, without falling or striding. When the TecnoBody PROKIN instrument detected LoS, the patient stood upright, feet at shoulder width, and looked straight ahead at the display. At the beginning of the test, the screen displays the target points in eight main directions (front, back, left, right, left front, right front, left rear, right rear) in turn, and the subject tilts their body quickly from the center to the target direction. The movement is maximized to make the display indicator icon appear close to the target point and record the percentage of the target reached by the patient, the body swing angle standard deviation, and time.
