# Supplementary material for: Effect of Subthalamic Nucleus Deep Brain Stimulation (STN-DBS) on balance performance in Parkinson's disease
Source: PLoS One. 2020 Sep 11;15(9):e0238936. doi: 10.1371/journal.pone.0238936 (PMC7486080; doi:10.1371/journal.pone.0238936)
Supplement: Attachment — Submitted filename: Response to Reviewers.docx [file pone.0238936.s004.docx]

Dear reviewers,

Thank you very much for giving us an opportunity to revise our manuscript. We appreciate the editor and reviewers very much for their constructive comments and suggestions on our manuscript entitled "Clinical experience of comprehensive treatment on the balance function of Parkinson's disease"(ID: MD-D-20-00182).

We have studied reviewers' comments carefully. According to the reviewer's detailed suggestions, we have made a careful revision on the orginal manuscript. All revised portions are marked in red in the revised manuscript. The corrections are as follows:

Reviewer #1: Major comments.

1.According to the title, the authors seem to explore effect of STN-DBS on balance performance in Parkinson's disease. But the introduction of the paper focus on analyzing effect of DBS and medicine on balance performance.(page 2, line 44-45).

Response:The introduction had been amendment carefully according to the comments.

2.The description of statistical analysis was too brief to understand. For example, authors should have clarified which data nonparametric tests were used to analysis ? Was one-way repeated ANOVA used to analysis effect of STN-DBS or medicine on balance performance wrong writen? Whether it ought to be “repeated ANOVA” or not? And why only one “F” or “P” presented in Table 2-4. We think “F” value result from STN-DBS group or Med-group separately. That is, whether the results indicated that DBS could significantly improve motor disability, balance performance, and Los in PD with repeated ANOVA, which is the same as Med-group? In conclusion, we think it is necessary to describe the method of statistical analysis in detail.

Response:The description of statistical analysis had been amendment carefully.

3.The discussion was long-winded written and lack of logic. Page 6, line 147-157 discussed pathology of Parkinson’s disease and balance dysfunction, which was repeatedly writen in later paragraphs. Page 7, line 180-182: the sentence “ Studies have shown that APR abnormalities in patients with PD are related to decreased acetylcholinergic content in the thalamus, and are not related to acetylcholine in the cortex and striatum dopamine.” may be meaningless in this section. The paper intended to study effect of STN-DBS on balance performance in Parkinson's disease, so we think it’s unnecessary to analyze effect of PPN-DBS stimulation in Page 7, line 183-192. Moreover, we think discussion in the paper is lacking of focus. Maybe authors more ought to analyze the meaning of the result, and the differences with previous researches. Whether the result is consistent with previous studies, if not, what possible factors may lead to the differents? And are there some lightspots in the paper?

4.Some important thesis in the paper prefer to add some supporting literatures. Ex, page 8, line 202-203, STN-DBS can also improve the central axis symptoms by reducing the abnormal inhibition of PPN activity by the STN or compensating for neuronal loss in the PPN.

Response:The discussion had been rewritten carefully according to the comments.

5.Authors seemed to only attributed the limitation of the study to the subjectivity of scale assessment. We think it may be unconscionable. As we know, this study was retrospective, which means the researcher could distinguish between the STN-DBS group and the Med group, leading to bias during assessing the scales. Moreover, there were also some confounding bias existing in the study deriving from retrospective study. In the other way, the sample size was small in the study, and it may lead to unstable result.

Response: the limitation of the study had been amendment.

6.The writing of the paper need to improve. There were some sentences hard to understand.For example, page 6,line 161-162, ”This is consistent with a previous study [8] and may be a result of the balance dysfunction in PD correlating with both the dopaminergic and cholinergic system.”. Page 8,line 214-215, “Therefore, the balance function in patients with DBS improved postoperation but was not accurately reflected due to the subjectivity and bias of the balance function score.” and so on.

Response:The paper had been rewritten carefully. Sentences and structure had been amendment logically.

Minor comments

1.Page 2, line 41-42: Whether the sentence ”Due to the fear of falling, the activities of patients with PD are restricted, which seriously affects their quality of life, and even life safety” was wrong described? That restricting activities of patients with PD would seriously affects life safety?

Response:The sentence had been amendment carefully according to the comments.

2.Page 2, line 45: “different treatment” ought to be “different treatments” . Response:The sentence had been amendment carefully according to the comments.

3.Page 4, line 89-90: “;” should be represented by “,”

Response:The sentence had been amendment carefully according to the comments.

4.Page 4, line 107: whether the “post-operation” represented “4 weeks after operation” or not?

Response:The sentence had been amendment carefully according to the comments.

5.Page 5, line 131: “We found no differences in the clinical characteristics of the DBS Group and Medicine Group” would be better to write as “We found no differences in clinical characteristics of the DBS Group and Medicine Group before operation.”

Response:The sentence had been amendment carefully according to the comments.

6.Page6, line 136: “The LEDD preoperation” ought to be “The LEDD in preoperation”

Response:The sentence had been amendment carefully according to the comments.

Reviewer #2: The authors performed a retrospective study of short term effects of STN-DBS stimulation on balance control. The issue is of importance in the field. A great strength of the study is that data from the surgery group are compared to data of a control, best medical treatment group. If correctly analyzed, such data would provide new and relevant information in the field. However, in the present version of the study report, there are major methodological weaknesses in data processing. In addition, the presentation of the methods and results needs to be thoroughly edited. The discussion needs to be focused on the reported data rather than on general considerations about the neuronal networks possibly involved in balance control and balance disorders in Parkinson’s disease patients. Overall, the paper needs serious editing and information needs to be presented in a more structured way. The abstract would also have to be rewritten accordingly.

Response:The abstraction, methods and results had been rewritten thoroughly edited accordingly.

Major concerns

* The specific variables recorded and analyzed need to be clearly presented and defined. Scoring needs to be explained. It is not clear when the first measure post-surgery was performed? (upon switching on stimulation a few days or weeks after surgery? At three months, after xx weeks of chronic stimulation and adjustments of parameter settings?)

Response:The specific variables recorded and analyzed had been amendment, scoring had been added clearly in the paper.

* It is said in the text that non parametric tests were used, but also that one-way repeated ANOVA were used. Which ANOVA? Friedman? Kruskall-Wallis? GLM? As Tables report F values, it seems that parametric ANOVAs were used. The statistical design to be precisely described. For each dependent variable, there were 4 time-point measures (preop, sometime early post-op, 6 months follow-up and 12 months follow-up) X 2 medication conditions (med off, med on). The most appropriate design (using parametric statistic) would thus be a GLM or ANOVA with a design 2 conditions X 4 time-points (repeated measures for this last factor). If non parametric tests are used, the design is more complex and requires 2 steps.

Response:The Statistical analysis had been amendment accordingly.

* Demographical data cannot be analyzed as the dependent variables of the study. The two groups need to be compared using t tests or a non-parametric equivalent.

Response:The Statistical analysis had been amendment accordingly.

* Data are presented as mean and standard deviation in the Tables, and median and quartiles in the text for some of them (age, PD duration) but not all (LEDD). For the reader, it would be friendlier to have a coherent way of presenting the data. Statistical outcomes for the demographical data need to be reported. One would also want to see the MDS-UPDRS III total score Med off and Med on for the two groups, before surgery (there are given in Tables 2 and 3, but the reader would want to see them with the demographical data, as they contribute to describing the population).

Response:The data presented had been amendment accordingly.

* In Table 1, it is said that males represented 8.5 and 8.4% of the sample; this means that 92% of the patients were females? This would be very unusual and would need to be discussed. In addition, 8.5% of 16 patients are 1.36 persons, and 8.4% of 20 patients are 1.68 persons. This does not make much sense. Examination of Table 1, also shows that disease onset was about 50 years of age in average in the DBS group, and about 45 years of age in the medical

Response:The data presented had been amendment accordingly.

treatment group. The latter is rather early onset. This should be discussed also.

* Regarding the dependent variables, Tables 2 and 3 report mean and SD, and a single F and p, without explanation of what these Fs and ps refer to: effect of group? Effect of time? What about interactions? Were they significant? If yes post-hoc analyses need to be reported.

Response:The data presented and statistical analysis had been amendment accordingly.

In the text, the outcome of the statistical analyses needs to be precisely reported. “No obvious differences” (page 6, lines 138-139) is not an objective way of reporting. Either there were statistically significant differences, or there were not. Report successively the outcome for each variable.

Response:The sentence had been amendment according to the comments.

Discussion

In its present form, the discussion does not discuss the results of the study. There are many general considerations on balance difficulties in PD, and potentially involved brain regions. The precise outcomes as well as characteristics of the sample and limitations of the study need to be specifically examined and interpreted.

Response:The discussion had been rewritten carefully. Sentences and structure had been amendment logically.

Minor concerns

Response: All the minor concerns had been amendment carefully according to the comments.

Introduction:

* what does “primary” PD means? Idiopathic? (page 2, line 35)

* What do you mean with “increases body swing” (page 2, line 37); please be specific in the description of the sign

* What does “decreases coordination” refer to? Which coordination are you referring to? (page 2, line 37)

* Aim of the study (page 2, line 44-45 “In this study, we retrospectively 45 analyzed patients treated by DBS and medicine to verify the effect of different treatment.”) is too unspecific and should be rephrased. Aim of the study seems to have been to compare the effect of medical treatment vs. STN-DBS on balance control in patients with PD.

Methods:

* Inclusion criteria: please make several sentences or a structured list to make the information legible (page 2 last line to page 3 line 57)

* What was the test and cut-off used to exclude patients with “obvious cognitive dysfunction”? (page 3, line 60); same issue for anxiety and depression.

DBS programming

* Page 4, line 85: contacts, not contactors

* Page 4, line 87: What does it mean to test the stimulation parameters “with attention on the … and drug side effects”? If testing the effect of the stimulation, the unwanted effects are related to stimulation parameters, not to drug.

* Page 4 lines 88-91. “Problems in balance….”. Delete this long sentence. This sentence refers to usual procedures of parameter are considerations on strategies that have been tried to improve balance or gait issues in patients with DBS. You may want to discuss these issues in the discussion, if some of the patients of the present study were actually stimulated in the SNr.

* Page 4 lines 98-103. Again these are general comments regarding management of patients after DBS implantation and is not really relevant to the present report. This paragraph should be deleted, except for the 1st and last sentences. (In addition, FOG can be induced by levodopa, so that increase in levodopa dose is not always the appropriate strategy for clinical management of FOG, especially in patients with dyskinesia.)

* Evaluation of quality of life, motor disability, balance performance and limit of stability (page 4 – lines 104 and following). This whole paragraph needs to be structured. Scales need to be presented (PDQ-39, item 3.12 of MDS-UPDRS III, BBS). Description of the LOS procedure needs editing to be fully understandable (for example, regarding the position of the patients, it would be simpler to state that patients were standing upright, feet at shoulder width, and looking straight ahead at the display. The task needs to be more clearly explained, as well as the variables (what is the “target body swing angle”?). There seems to be a contradiction between performing accurate movements to the target and maximizing movement. “Small limbs” are called “upper limbs” (I suppose).

Thanks for your thoughtful suggestion. I really appreciate that 2 reviewers spend a lot of time and energy on this paper. Your thoughtful comments helped me to present our study structurally and logically. We thought carefully about every word and rewrite the paper.

Kind regards,

Haitao Li, M.M.

Corresponding author: Xiaoguang Tong

E-mail address: tongxghh@163.com
