# Supplementary material for: Effect of Subthalamic Nucleus Deep Brain Stimulation (STN-DBS) on balance performance in Parkinson's disease
Source: PLoS One. 2020 Sep 11;15(9):e0238936. doi: 10.1371/journal.pone.0238936 (PMC7486080; doi:10.1371/journal.pone.0238936)
Supplement: Attachment — Submitted filename: letter.docx [file pone.0238936.s005.docx]

Reviewer #1: Major comments.
1.Specific statistical methods could be described in clinical characteristics of the subjects, instead of nonparametric tests briefly In Page 5, line 128-129.

Response: The statistical methods had been rewrite in detail.
2.Discussion of the article still lack of attractive. The author could discuss the results more. The detailed characteristics, advantages and clinical significance of this study could be discussed. Moreover, there could have some other mechanisms about the improvement of balance performance in STN-DBS, but not only for the improvement of bradykinesia or rigidity. And limitations of the study could be discussed in detail. In general, the discussion of the article was not well-written.

Response: Thanks a lot for your comments. We rewrite the discussion section according to your comments. The clinical significance and limitation of this study had been added into discussion.
Minor comments
1.The additional instructions In Page 4-5, line 101-113 were not supposed to be described in the main body of the article.

Response: The additional instructions had been moved to end of the article.
2. In Page 2, line 36-38, “Balance dysfunction is one of the causes of posture instability, decreases motor coordination, and becomes more and more serious as PD progresses, and has been identified as one of the independent risk factors for falls.” The state was wrong written.
3. In Page 2, line 43, “treatment” could be “treatments”.

Response: It had been amendment according to the comment.
4. In Page 6, line 150, “of” could be “or”.

Response: It had been amendment according to the comment.
5. In Page 6, line 152-153, what does the author mean by “ different studies have different criteria“?

Response:In this sentence, we are trying to say that different including criteria might lead to diverse outcomes in different study. We rewrite the sentence to make it clearly.
6. In Page 7, line 180, “ which could inhibit the excitability of the SNr/GPi”. Whether STN-DBS would inhibit the excitability of the GPi or not?

Response:In this sentence we are trying to say that STN-DBS would inhibit the excitability of the GPi. It had been amendment in the article.

Reviewer #2: The revised version is much improved and the authors strived to answer most of the comments made on the original version.
In addition to serious editing for the English language and syntax, their remains some questions and minor issues.

Thanks a lot for your help in review my article. I really appreciate it.
Abstact
Line 20. “One-way ANOVA” needs to be corrected as the statistical design was repeated measures.

Response: It had been amendment according to the comment.
Introduction
Line 45. “In this study we retrospectively analyzed patients to verify the effect of STN-DBS on balance performance.”
The study actually included a group with best medical treatment suggesting that one aim was also to compare STN-DBS and best medical treatment. In the present version, data are presented as parallel analyses in the two groups. There is no direct comparison between the two groups but their alternation in the Tables. However, there is little point in including a group with best medical treatment if not to compare the two procedures. I would therefore suggest to indicate the comparison here and use a statistical design enabling direct comparison.
Response: thank you for your suggestion in the Statistical analysis.In addition, we analyzed the motor disability and balance function between STN-DBS group and Medicine group. It had been amendment according to the comment.
Methods
Line 56. Third inclusion criterion “MDS-UPDRS III score improvement >30% in acute levodopa drug test”. For the STN group, it should be mentioned that this was expected before surgery. If the criterion also applies after surgery, it needs to be justified and stated, and it should be mentioned whether this in Stim On or Stim Off.

Response: For the STN group, acute levodopa drug test was expected before surgery.It had been amendment according to the comment.
Line 86, BDS programming. “During programming, the limb with severe symptoms was programmed first”. Was it the limb or the side? One supposes it is “with the more severe symptoms”?

Response: It should be “the limb side with more severe symptoms”.It had been amendment according to the comment.

Line 96. Post-op first assessment. Please indicate the delay between the time stimulation was switched on and that of the assessment. Was it immediately or within hours? After a day or two?

Response: “the evaluate were underwent in 4 hous after the DBS turned on”.It had been amendment according to the comment.
Line 101. Explain the scoring of the PDQ-39 (whether higher score reflects worse or better quality of life).

Response: “higher score means worse quality of life.”It had been amendment according to the comment.
Line 104-105. “The evaluation refers to the functional state of the patient who is currently taking anti-Parkinson drug with the best efficacy status.” This is not clear. The MDS-UPDRS is performed both Off and On Med. Under the Off Med condition, the patient has not taken any drug. What does “the best efficacy status” mean?
It is indicated that the same dose of medication was used before and after surgery. Was that the usual morning dose or were the assessments run during a Levodopa challenge (using a greater medication dose)?

Response: This part of article had been rewrite according to the comment.
Line 120. “The movement is maximized to make the display indicator….” What the authors mean to say is not clear.

Response: This part of article had been rewrite according to the comment.
Lines 127-131. Statistical analysis.
Demographical data. Only the data at inclusion should to be reported in Table 1 to demonstrate that the two groups did not differ before the study started.
LEDD, MDS-UPDRS III, PDQ-39 are dependent variables with repeated measures and need to be analyzed with a relevant statistical design (Anovas with repeated measures of GLM with repeated measures, as for the other dependent variables).
For all variables, the statistical design should be reported (As mentioned above, I would suggest ANOVA or GLM with 2 Group X 4 Time-points with repeated measures for the last factor). Tables 2 and 3 suggest that two different ANOVAS were run for the DBS group and for the Medicine group. Such design does not enable comparison between the two groups, leading to questioning why a best medical treatment group was included.
Response: We analyzed the motor disability and balance function between STN-DBS group and Medicine group. It had been amendment according to the comment.

Line 132 and below. Results section.
Provide all statistical outcomes. Presumably, with a 2 Group X 4 Time-points design, there should be either an effect of Group, or an effect of Time-point, and an interaction between these two factors. Post-hoc comparisons need to be reported.
Table 3 shows a trend for improvement of MDS-UPDRS III in the Medicine group under the Med On condition. This trend should be discussed.
Table 4 shows trends for improvement of swing angle SD and time in the Medicine group under the Med Off condition. Could this be a learning effect? It should be discussed.

Response: We analyzed the motor disability and balance function between STN-DBS group and Medicine group. It had been amendment according to the comment.

Discussion

Response: Thank you very much for your comments with this manuscript. I am grateful for your help in reviewing this paper. The discussion section had been rewrite carefully according to the comments.

Line 154. “In this study patients with H&Y3 stage were included, and certain results were obtained…” “certain results” is too vague.
Line 165-167. “Moreover, due to compensation with a long duration of bradykinesia, rigidity and tremor, the balance dysfunction may be aggravated when rigidity is released by medicine.” Please elaborate the argument.
Line 168. The second sentence is missing its end.

Line 179-180. The mechanisms of action of DBS are still much debated. The authors should be less assertive.
Line 184-185. Regarding balance, Thevathasan et al., 2018, would be a relevant reference.
Line 188. It is not always true that STN-DBS improves camptocormia. In fact, it tends to have no effect (Debû et al., 2018) especially if surgery is performed more than 18 months after camptocormia installation (Schulz-Schaeffer et al., 2015).
Regarding the discussion in general, a number of other studies have previously concluded that STN-DBS does improve balance in the short term (1-3 years) but that balance dysfunction then increases again with disease progression. This should be mentioned in the discussion, and the authors should also elaborate some more on the new information provided by their study, esp. regarding the interest of using objective measures of balance (while clinically, however, the patients get progressively worse and fall more often, which suggests that clinical maneuvers are not so bad to assess postural stability).
